# Supplementary material for: Microclimate buffering and thermal tolerance across elevations in a tropical butterfly
Source: J Exp Biol. 2020 Apr 16;223(8):jeb220426. doi: 10.1242/jeb.220426 (PMC7174841; doi:10.1242/jeb.220426)
Supplement: Supplementary information [file jexbio-223-220426-s1.pdf]

Table S1. Localities of the four areas in Ecuador under study and summary information. Coordinates and altitudes are averaged across the seven sites in each area. Altitude represents meters above sea level. Mean annual precipitation was obtained from WorldClim2 (Fick and Hijmans 2017). Nearby towns/ reserves: 1) Highlands West, Mashpi Lodge (Pichincha), 2) Lowlands West, Tortugo (Pichincha), 3) Highlands East, Reserva Narupa (Napo), 4) Lowlands East, Reserva Jatun Satcha (Napo). Positive values indicate WorldClim2 is overestimating forest temperatures.

Altitude	Andes slope	Lat.	Long.	Altitude (m.a.s.l.)	WC2 annual precipitation	Annual mean temperature (°C)			WorldClim2 T_{mean} deviation	
						understory	canopy	WorldClim2 mean (BIO1)	WC2 – understory	WC2 – canopy
High	West	0.16	-78.86	1208.7	2845	19.01 ± 0.13	19.22 ± 0.18	20.2	+1.2	+1.0
Low	West	0.21	-78.95	468.4	2623	22.66 ± 0.14	22.94 ± 0.13	23.1	+0.4	+0.2
High	East	-0.69	-77.75	1219.9	4040	19.09 ± 0.1	19.3 ± 0.14	19.3	+0.2	0
Low	East	-1.07	-77.63	420.7	3746	23.52 ± 0.07	23.99 ± 0.11	23.6	+0.1	-0.4

Journal of Experimental Biology • Supplementary information

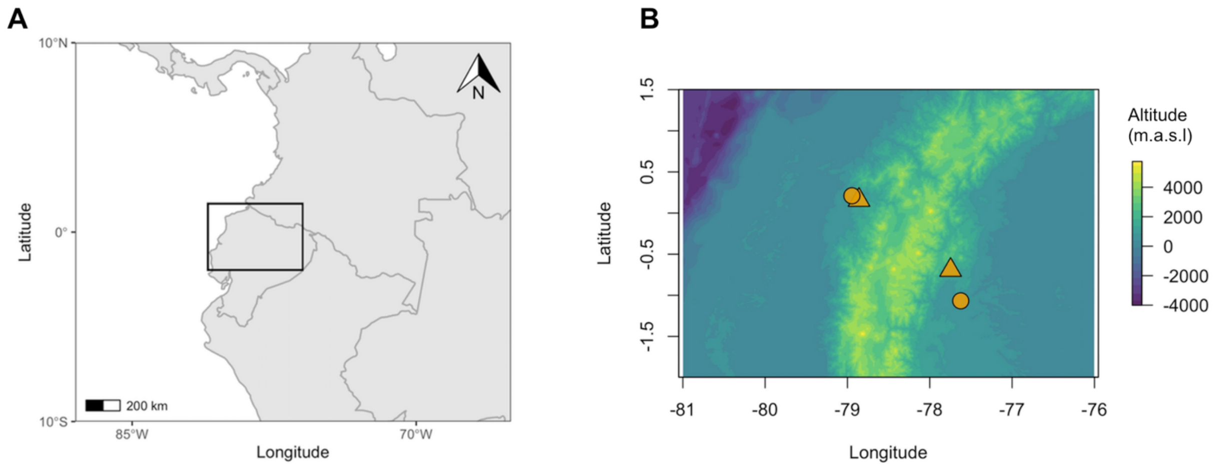

Figure S1. Microclimate sampling sites in Ecuador along both sides of the Andes. A) Western South America, rectangle represents area mapped in B. B) Elevation map of area under study with high altitude (triangles) and low altitude (circles) areas. In each area there are seven plots with paired canopy and understory temperature data loggers and humidity dataloggers for two of the seven plots.

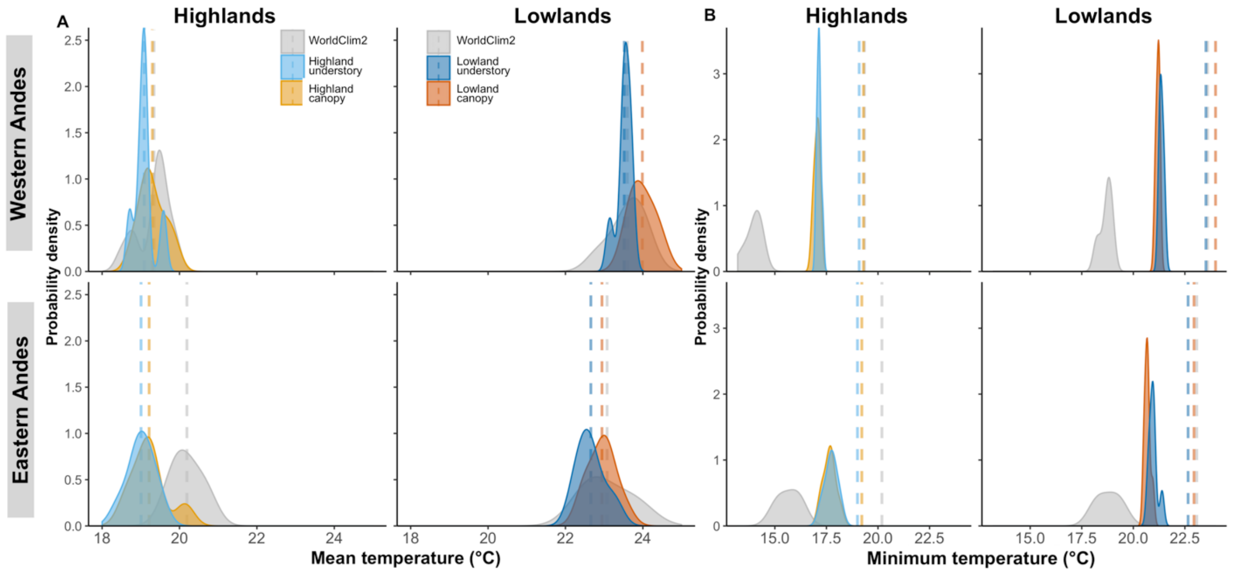

Figure S2. Daily mean temperature (A) and mean daily minimum temperature (B), obtained mean yearly logger daily mean and minimum temperatures and compared them to WorldClim2 interpolated daily mean and minima (T_{avg}, T_{min}) for these areas. Vertical dashed lines represent means per group.

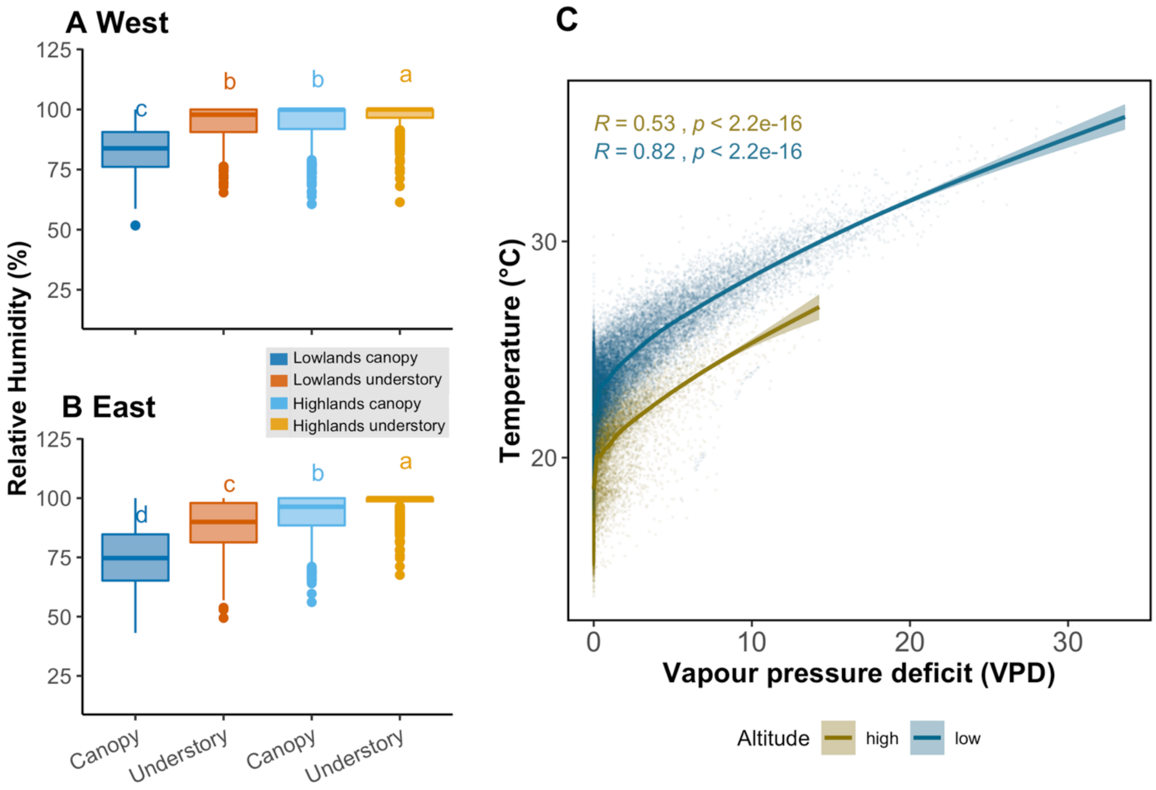

Figure S3. A), B) Daily minima relative humidity across microclimates and elevations. The bottom and top of the boxes represent the first and third quartiles, respectively, the bold line represents the median, the points represent outliers, and the vertical line delimits maximum and minimum non-outlier observations. Same superscripts represent no significant differences (Tukey post-hoc test p>0.05). C) Vapour pressure deficit relationship with temperature across elevations (high=gold, low=blue). Lines are fitted generalised additive models and shaded areas confidence intervals. Each point represents a single record.

Journal of Experimental Biology • Supplementary information

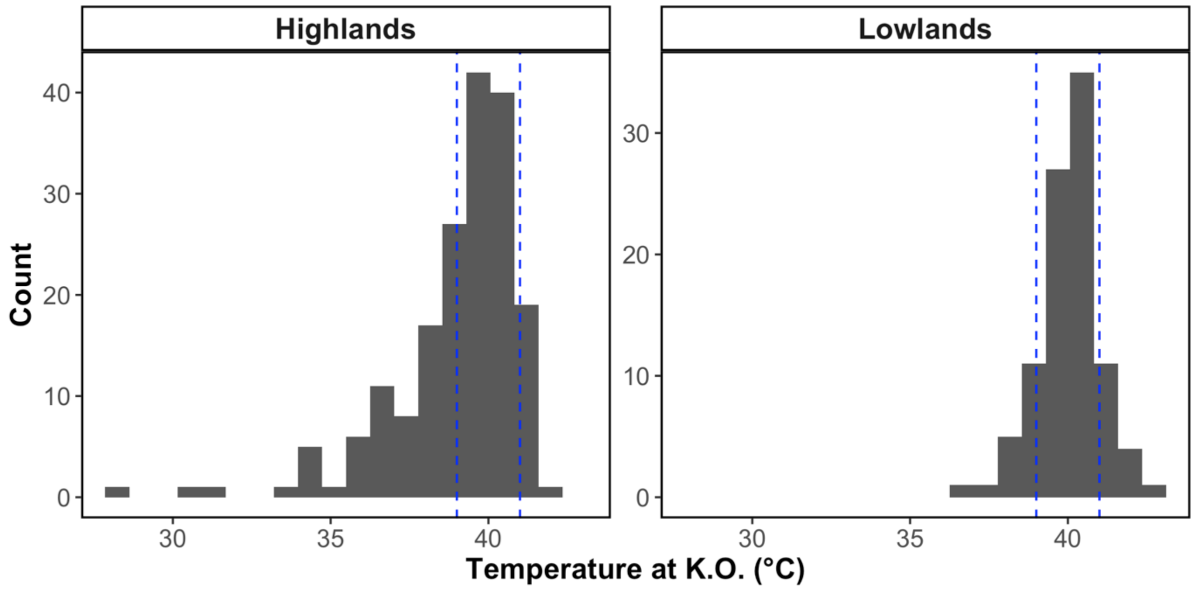

Figure S4. Variation in temperature at K.O. in wild heat knockdown experiments across ten species. We aimed for chambers to be at a constant temperature between 39C and 41C (delimited by the dashed blue lines), but in the highlands many individuals got knocked down before the temperature chamber reached 39C (left-skewed tail, left plot).

Journal of Experimental Biology • Supplementary information

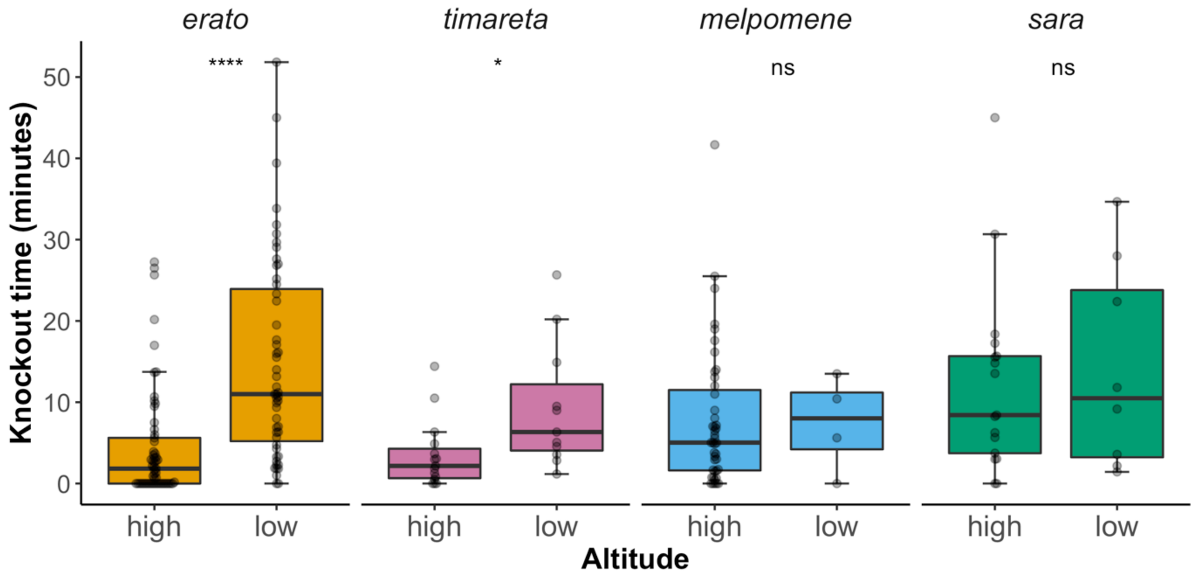

Figure S5. Within species variation in heat knockout time in wild individuals. The bottom and top of the boxplots represent the first and third quartiles, respectively, the bold line represents the median, and the points represent all values. Stars represent significance levels of two sample t-tests between high and low altitude populations (*< 0.05, **<0.01, ***<0.001, ns not significant).

Journal of Experimental Biology • Supplementary information

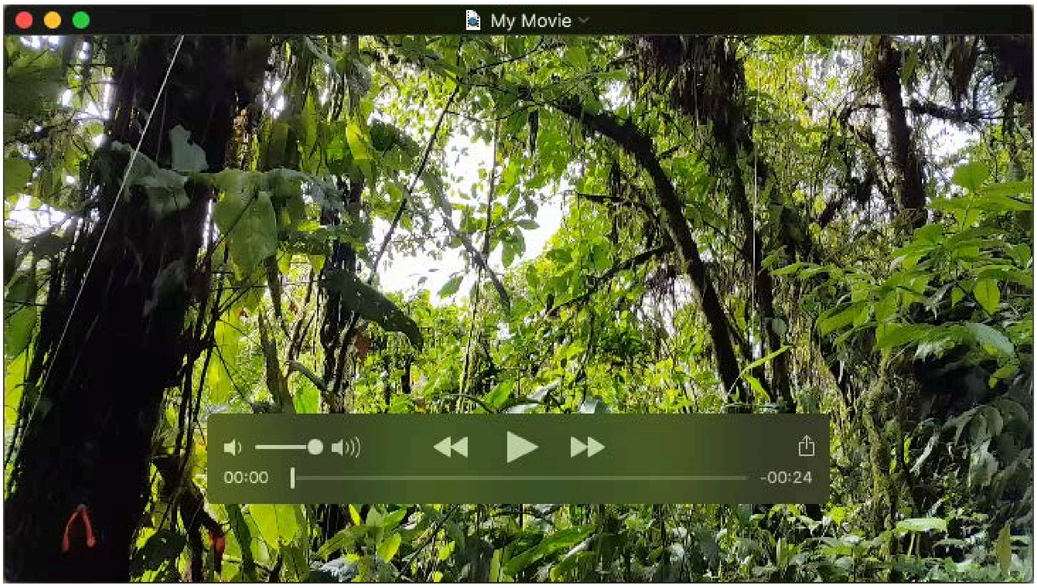

Movie 1. Temperature data logger being hoisted up to the subcanopy with nylon thread in the western highlands of Ecuador (Mashpi Reserve).
